# *Mycobacterium orygis* Lymphadenitis in New York, USA

**DOI:** 10.3201/eid2310.170490

**Published:** 2017-10

**Authors:** Luis A. Marcos, Eric D. Spitzer, Rahul Mahapatra, Yupo Ma, Tanya A. Halse, Joseph Shea, Michelle Isabelle, Pascal Lapierre, Vincent E. Escuyer

**Affiliations:** Stony Brook University, Stony Brook, New York, USA (L.A. Marcos, E.D. Spitzer, R. Mahapatra, Y. Ma);; Wadsworth Center, New York State Department of Health, Albany, New York, USA (T.A. Halse, J. Shea, M. Isabelle, P. Lapierre, V.E. Escuyer)

**Keywords:** mycobacterium orygis, antelope, waterbuck, rhesus monkey, oryx, lymphadenitis, lymphoma, genome, New York, United States, USA, Rv2042c, G1113A, *gyrB* gene, bacteria, tuberculosis and other mycobacteria

## Abstract

We report a case of lymphadenitis caused by *Mycobacterium orygis* in an immunocompetent person in Stony Brook, New York, USA. Initial real-time PCR assay failed to provide a final subspecies identification within the *M. tuberculosis* complex, but whole-genome sequencing characterized the isolate as *M. orygis*.

Genomic analysis has previously shown that the *Mycobacterium tuberculosis* (MTB) complex comprises >8 distinct subgroups: *M. tuberculosis*, *M. africanum*, *M. canettii*, *M. bovis*, *M. caprae*, *M. pinnipedii*, *M. microti*, and *M. mungi* (*1*). *M. orygis* was first characterized in Africa and South Asia in 2012 based on examination of 22 isolates selected for the similarity of their IS6110 restriction fragment length polymorphism patterns to previously described oryx bacilli (*2*). Eleven of these isolates were from animals (a cow, a rhesus monkey, and types of antelope including oryx), and 11 were from humans (9 from South Asia). On the basis of single-nucleotide polymorphism (SNP) and region of difference (RD) analysis, van Ingen et al. concluded that these mycobacteria belonged to a phylogenetically distinct lineage of the clonal MTB complex (*2*). *M. orygis* is also distinguished by a mutation in gene Rv2042c (*2*) and a G1113A mutation in the *gyrB* gene (*3*).

We report a case of lymphadenitis caused by *M. orygis* in an immunocompetent person in Stony Brook, New York, USA. During July 2015, we diagnosed pneumonia in the upper lobe of the right lung in a woman, 71 years of age, who had a remote history of lymphoma. The condition was characterized by enlarged lymph nodes. The patient was born in Pakistan, moved to India at age 1, and emigrated to the United States ≈25 years before onset; her preimmigration TB skin test was <5 mm (bacillus Calmette-Guérin vaccinated), and chest radiograph results were negative. She drank unpasteurized milk while living in India.

We completed positron emission and computed tomography scans by using intravenous F-18 fluoro-2-deoxyglucose that detected hypermetabolic foci in the right axilla, subpectoral, subcarinal, and para hilar regions. QuantiFERON-TB Gold in-tube system (Quest Diagnostics, Inc., Lyndhurst, NJ, USA) test result was positive (TB antigen minus nil value 3.58 IU/Ml, mitogen minus nil value 8.5 IU/mL). Three induced sputum samples for acid-fast bacilli smear and cultures were negative. Because of the patient’s history of lymphoma, we biopsied the subpectoral lymph node. Histopathology revealed diffuse large caseating granulomas with extensive central necrosis, small lymphocytes, plasma cells, and histiocytes. Grocott's Methenamine Silver Stain (Ventana Medical Systems, Inc., Tucson, AZ, USA), and acid-fast bacilli stains did not detect organisms. Bacterial and fungal cultures were negative.

Mycobacterium Growth Indicator Tube (MGIT) system turned positive on day 29, and the isolate was identified as MTB complex by probe hybridization (Hologic., Inc, San Diego, CA, USA). Further testing at the New York State Department of Health with real-time PCR using primers/probes specific for 4 MTB regions of difference yielded an inconclusive pattern (*4*). Results were positive for RD1 and RD4, but negative for RD9 and RD12; subspecies identification was initially reported as “inconclusive” because this pattern did not match the signature patterns used to determine MTB complex species with this assay.

We performed a whole-genome sequencing assay that confirmed the absence of RD9 and RD12 and identified the isolate as *M. orygis*, as reported by Shea et al (*5*). This isolate belonged to Spogliotype International Type 587, contained the specific *gyrB* SNP at position 1113 (*3*), and lacked resistance-associated mutations, suggesting susceptibility to all tested current antituberculosis agents. The patient received first-line, 4-drug therapy.

*M. orygis* infections in humans have been rarely reported. In Australia, of 1,763 case-patients diagnosed with MTB complex infection, 8 causative pathogens were identified as *M. orygis*; all of the patients were born in India (*6*). In New Zealand, Dawson et al. used advanced molecular techniques to demonstrate a transmission of *M. orygis* from a human, who emigrated from India, to a cow (*7*).

*M. orygis* infection may be underreported in the literature because cases may be identified as MTB complex or misidentified as *M. africanum* or *M. bovis* (*8*). Through whole-genome sequencing, the New York State Department of Health identified 8 additional cases of *M. orygis* of 6,322 MTB complex isolates from New York tested (3 pulmonary, 2 lymph node, and 2 abscess samples) that were received during 2005–2016 but were initially misidentified (data not shown). All patients were from India, Pakistan, or Nepal and had moved to the United States. SNP analysis indicated that the *M. orygis* isolates were genetically similar, but all were distant from other members of the MTB complex (Figure) and contained the G1113A mutation in *gyrB*. The number of SNPs separating the 8 *M. orygis* isolates was 106–323, which excludes their belonging to an epidemiologic transmission cluster (*9*) and strongly suggests that the infections were independently acquired.

**Figure:**
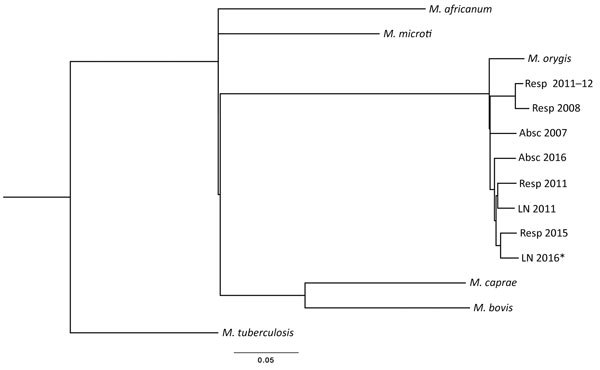
Maximum-likelihood single-nucleotide polymorphism (SNP) tree of 8 *Mycobacterium orygis* and 1 *M. caprae* isolates obtained from patients in New York, USA. Alignment of 5,242 total SNP positions was calculated by using PhyML version 20111216 (http://www.atgc-montpellier.fr/phyml/) general time reversible plus gamma model under 8 categories with best of nearest-neighbor interchange, subtree pruning, and regrafting with 5 random starting trees. Included in the tree are *M. tuberculosis* H37Rv (GenBank accession no. NC_000962), *M. orygis* (accession no. APKD01000001.1), *M. bovis* (accession no. NC_002945.3), *M. africanum* (accession no. NC_015758.1), and *M. microti* (ATCC 35782) reference sequences. SNP analysis of the isolate from the patient described in this study (LN 2016) with 7 other *M. orygis* strains identified at the New York State Department of Health showed differences ranging from 170 (closest) to 323 (farthest) SNPs. By comparison, the closest non–*M. orygis* *M. tuberculosis* complex species is *M. microti* (1,880 SNPs). All *M. orygis* strains are grouped with 100% bootstrap support. Scale bar indicates average number of substitutions per site. Specimen sources: Resp, respiratory; Absc, abcess; LN, lymph node (BioProject ID PRJNA389109 containing BioSample accessions SAMN07190143–50).

We found no previous reports of *M. orygis* originating in the Americas; the most notable epidemiologic risk factor in this patient was prior residency in India, where *M. orygis* was found in a variety of animals (*10*). Because all organisms in the MTB complex have a distinct host preference, it is possible that *M. orygis* is mostly present in animals and few cases occur in humans, similar to *M. bovis*. This case demonstrates the value of molecular methodologies such as whole-genome sequencing for providing more detailed insight into the clinical and epidemiologic aspects of the MTB complex.
